# Transparent Cellulose/Multi-Walled Carbon Nanotube Hybrids with Improved Ultraviolet-Shielding Properties Prepared from Cotton Textile Waste

**DOI:** 10.3390/polym16091269

**Published:** 2024-05-01

**Authors:** Zhen Xu, Yingying Ma, Xiaohui Yao, Hongxu Wang, Qian Zhang, Qiance Ma, Zhanrui Zhang, Guangmei Xia, Jinming Zhang, Fengshan Zhang

**Affiliations:** 1 Key Laboratory of Pulp and Paper Science & Technology of Ministry of Education, State Key Laboratory of Biobased Material and Green Papermaking, Faculty of Light Industry, Qilu University of Technology (Shandong Academy of Sciences), Jinan 250353, China; xz521953@qlu.edu.cn (Z.X.); 17860212773@163.com (Y.M.); 19707066261@163.com (X.Y.); wanghongxu6320@163.com (H.W.); m15095097700@163.com (Q.Z.); maqianc23@163.com (Q.M.); zzr_419@163.com (Z.Z.); 2 Shandong Huatai Paper Co., Ltd. & Shandong Yellow Triangle Biotechnology Industry Research Institute Co., Ltd., Dongying 257000, China; 3 Beijing National Laboratory for Molecular Sciences, CAS Key Laboratory of Engineering Plastics, Institute of Chemistry, Chinese Academy of Sciences (CAS), Beijing 100190, China; zhjm@iccas.ac.cn

**Keywords:** cellulose films, multi-walled carbon nanotubes, transparency, anti-ultraviolet, cotton textile waste

## Abstract

Plastics offer many advantages and are widely used in various fields. Nevertheless, most plastics derived from petroleum are slow to degrade due to their stable polymer structure, posing serious threats to organisms and ecosystems. Thus, developing environmentally friendly and biodegradable plastics is imperative. In this study, biodegradable cellulose/multi-walled carbon nanotube (MCNT) hybrid gels and films with improved ultraviolet-shielding properties were successfully prepared using cotton textile waste as a resource. It was proven that MCNTs can be dispersed evenly in cellulose without any chemical or physical pretreatment. It was found that the contents of MCNTs had obvious effects on the structures and properties of hybrid films. Particularly, the averaged transmittance of cellulose/MCNT composite films in the range of 320–400 nm (T_320–400_) and 290–320 nm (T_290–320_) can be as low as 19.91% and 16.09%, when the content of MCNTs was 4.0%, much lower than those of pure cellulose films (T_320–400_: 84.12% and T_290–320_: 80.03%). Meanwhile, the water contact angles of the cellulose/MCNT films were increased by increasing the content of MCNTs. Most importantly, the mechanical performance of cellulose/MCNT films could be controlled by the additives of glycerol and MCNTs. The tensile strength of the cellulose/MCNT films was able to reach as high as 20.58 MPa, while the elongation at break was about 31.35%. To summarize, transparent cellulose/MCNT composites with enhanced ultraviolet-shielding properties can be manufactured successfully from low-cost cotton textile waste, which is beneficial not only in terms of environmental protection, but also the utilization of natural resources.

## 1. Introduction

Plastics have a wide range of applications in modern society as packaging and protective materials for drugs, food, and electronics due to their durability, good transmittance, high flexibility, light weight, and low cost. Nevertheless, most of the commercial plastics such as polyolefin, polyvinyl chloride, and polyethylene terephthalate are synthetic polymers that are derived from fossil fuels. It is reported that approximately 360 million tons of plastics are produced worldwide each year and more than 50% of them are thrown away after use [1,2,3,4]. These single-use plastics are smashed and accumulate in aquatic and terrestrial environments. It is reported that it will take hundreds of years for these synthetic plastics to degrade due to the stability of their carbon skeleton [5]. Not surprisingly, plastic pollution is ubiquitous in oceans and may cause great damage to ecosystems [4]. Particularly, micro-plastics with a size smaller than 5 mm can be easily consumed by, and accumulate within, living organisms in aquatic environments over time [2,6]. Consequently, micro-plastics can invade the human body through the food chain, leading to a great threat to human health [5,7,8]. Hence, developing biodegradable bio-based materials to replace these petroleum-based films is urgent and meaningful.

In fact, a series of bio-based materials manufactured from synthetic polymers, such as ester containing the polymers poly (butylene succinate), polyhydroxyalkanoate, and polylactic acid, or natural polymers composed of cellulose, chitosan, starch, and protein, have been developed [9,10]. In particular, natural polymers have been intensively investigated due to their inherent advantages of nontoxicity, biodegradability, abundance, and renewability. As the most abundant natural resources on earth, cellulose-based materials have increasingly attracted attention, and they are recognized as the most promising alternative to synthetic polymer films [11,12]. Nowadays, cellulose-based films show great potential in electrical devices, separation membranes, packaging materials, and light management materials due to their good transparency and mechanical performance [12,13]. However, neat cellulose films lack ultraviolet (UV)-shielding properties, which impedes their further application and development. As we know, organic polymer materials and human skin are susceptible to UV radiation [14,15]. Moreover, it has been clarified that prolonged exposure to UV rays may have harmful effects on the eyes, skin, and immune system of humans. Additionally, UV-shielding materials with good visible light transmittance represent a hot topic in the scientific community over recent decades. Generally, the UV-shielding ability of materials can be determined by additives. Therefore, it is of great significance to exploit cellulose-based materials for sunscreen ingredients.

Anti-ultraviolet agents are generally divided into inorganic UV blockers (e.g., SiO_2_, ZnO, TiO_2_, and carbon black) and organic UV absorbers (natural dyes, salicylate, benzophenone, and benzotriazoles derivatives) according to their anti-ultraviolet mechanism [14,16]. It is worth noticing that some novel anti-ultraviolet substances have been developed recently to prepare polymer films with UV-shielding performance. For example, as a new emerging nano-building unit, aramid nanofibers (ANFs) are derived from macro-scale fibers, displaying many advantages, and have been added to various polymer matrixes. Cellulose/ANF films with an improved ultraviolet-shielding capacity have been reported by researchers [17,18]. Meanwhile, as a bio-degradable bio-based UV screening agent, lignin demonstrates broad prospects for anti-ultraviolet applications. Regenerated lingo-cellulosic films with effective UV-shielding capacity were successfully fabricated from agricultural and domestic solid waste [19]. Furthermore, anti-ultraviolet cellulose nanofibers and lignin hybrid films can be obtained via casting or vacuum filtration methods [20,21]. To alleviate the poor compatibility of lignin in polymer matrixes, lignin can be prepared into nanoparticles via hydroxymethylation, acidolysis, and nano-precipitation approaches [22,23]. In addition, graphene oxide sheets with an oxygen-containing edge and graphitic structural core display promising potential in absorbing UV rays and have been composited with poly(vinyl alcohol) (PVA) [14], cellulose [15], and polyurethane (PU) [16,24] to prepare hybrid films. Ultraviolet protection polyethylene terephthalate and cotton fabrics have also been created by depositing graphene oxide [25,26,27], but developing new UV screening agents is still necessary.

As one-dimensional hollow cylindrical nano-materials, carbon nanotubes (CNTs) with excellent stability, optical, mechanical, and electrical properties have been incorporated into various organic matrixes to strengthen their electromagnetic, barrier, thermal, electrochemical, mechanical, and dielectric properties, demonstrating a range of applications in many fields [12,28,29,30,31,32,33]. It has been found that carbon nanotubes have been employed in super-capacitor electrodes and the CNT-based nanocomposites have mechanical resistance, desirable structure, and high surface area [28,34]. Meanwhile, CNT-based composites show efficient electromagnetic-interference-shielding capacity due to their high magnetism, electrical conductivity, aspect ratio, real/imaginary permittivity, and interfacial polarization [35,36]. Moreover, carbon nanotubes offer excellent electron affinity and transportation performance in some special polymers, which can be applied in bulk to heterojunction solar cells [28]. Additionally, carbon nanotubes have been adopted in water purification [1], nanocomposite sensors [28], thermoelectric devices [37], and cancer treatment [29,30], due to their excellent performance. However, the optical performance of carbon nanotubes currently seems to attract less attention.

In this research, transparent cellulose/multi-walled carbon nanotube (MCNT) hybrid gels and films with improved ultraviolet-shielding properties were fabricated successfully using the sol–gel approach described in “Fabrication of cellulose/MCNT composites”. On one hand, it is difficult to disperse carbon nanotubes (CNTs) evenly in a polymer matrix, as CNTs are hydrophobic and easy to aggregate, but the homogeneity of carbon nanotube composites is important to their structure and properties. Room-temperature ionic liquids (RTILs) are a type of powerful solvent, and it has been reported that a homogeneous single-walled carbon nanotube (SWCNT)/RTIL dispersion was achieved after grinding these mixtures without adding any other additives [38,39]. Therefore, one kind of RTIL, 1-allyl-3-methylimidazolium chloride (AmimCl), was used to disperse MCNTs, and multi-walled carbon nanotubes were dispersed uniformly in the ionic liquid 1-allyl-3-methylimidazolium chloride (AmimCl) by mechanical stirring without any chemical or physical pretreatments. On the other hand, AmimCl is also one of the non-derivative solvents for cellulose, and the pretreated waste cotton textiles can be dissolved efficiently in AmimCl according to our previous work [18]. Then, the MCNT/AmimCl dispersion was mixed in situ with cellulose/AmimCl solution. Finally, a homogeneous cellulose/MCNT/AmimCl mixed dispersion was obtained and could remain stable for more than 11 months due to the strong hydrogen bonding and cation-π interactions of the components. Moreover, the optical, thermal, and mechanical performances of the cellulose/MCNT composite films with different proportions of MCNTs were also investigated systematically. In a short time, homogeneous and transparent cellulose/multi-walled carbon nanotube hybrids with improved ultraviolet-shielding properties were successfully prepared from the cotton textile waste in this study.

## 2. Materials and Methods

### 2.1. Materials and Chemicals

Pretreated waste cotton textiles (p-WCTs) were used as a cellulose resource and collected from households [18]. Multi-walled carbon nanotubes (MCNTs) with high purity (>95%) were bought from Aladdin. The outside diameter and length of MCNTs were about 8–15 nm and 50 μm, respectively. Deionized water was self-produced in lab. 1-allyl-3-methylimidazolium chloride (AmimCl), one type of room-temperature ionic liquid, was donated by Shandong ICCAS-Henglian Biobased Materials Co., Ltd. (Weifang, China) and used without further purification [40].

### 2.2. Fabrication of Cellulose/MCNT Composites

**Figure 1:**
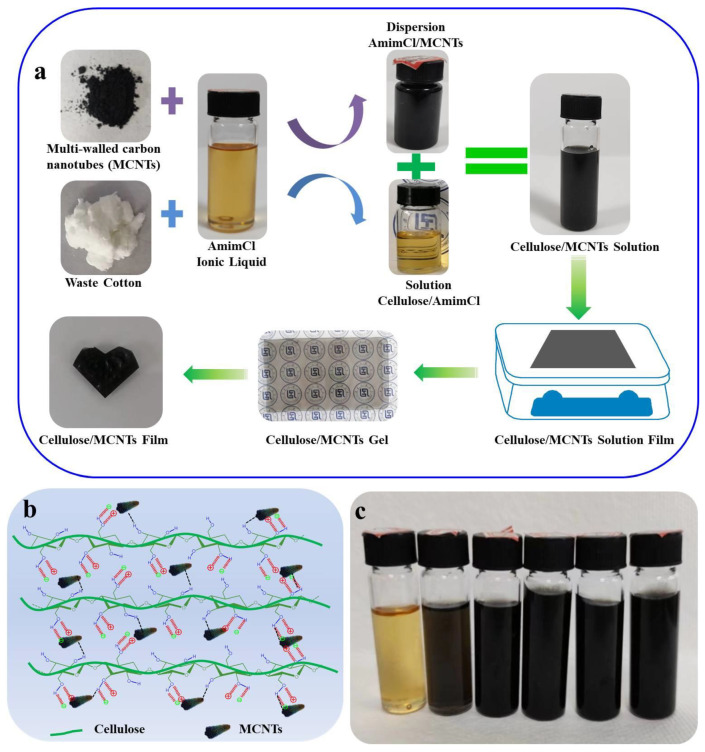
(**a**) Preparation process of cellulose/MCNT composite solutions, hydrogels, and films; (**b**) interactions between cellulose chains and MCNTs; (**c**) digital picture of cellulose/MCNT composite solutions after preparation for more than 11 months.

The preparation process for cellulose/MCNT composite solutions, gels, and films is exhibited in Figure 1a. Firstly, the pretreated waste cotton textiles (p-WCTs) were shredded and dissolved into AmimCl under mechanical stirring at 80 °C for 4 h to obtain 2.0 wt% cellulose solutions. Meanwhile, MCNTs with a mass ratio of 2.0% were dispersed homogeneously into AmimCl by vigorous mechanical stirring. Subsequently, the MCNT dispersion and cellulose solution were mixed together in situ and mechanically stirred for 40 min. Then, about 50 g mixture was poured onto the glass plate and formed 1000 μm of liquid film, and then the plate with liquid film was put into the deionized water to prepare cellulose/MCNT hydrogels via sol–gel technology. It is worth highlighting that the last coagulation bath of the prepared cellulose/MCNT hydrogels was deionized water with 8.0 wt% of glycerol. Finally, cellulose/MCNT hydrogels were further dried by Kessel paper dryer at 97 °C for 6 min to achieve C-MCNT0, C-MCNT0.5, C-MCNT1.0, C-MCNT2.0, C-MCNT3.0, and C-MCNT4.0 films, where 0.5, 1.0, 2.0, 3.0, and 4.0 was the mass ratio of MCNTs in the dried film samples, as displayed in Table 1.

### 2.3. Characterization

#### 2.3.1. Ultraviolet and Visible (UV-Vis) Spectra of the Cellulose/MCNT Composites

The transmittance and absorbance spectra of cellulose/MCNT composites ranging from 200 nm to 800 nm were recorded by UV 2600 Ultraviolet spectrophotometer (Shimadzu, Japan). Meanwhile, the averaged transmittance in the range of 320–400 nm (T_320–400_) and 290–320 nm (T_290–320_) was calculated and adopted to judge the ultraviolet-shielding properties of the cellulose/MCNT composites.

#### 2.3.2. Wide-Angle X-ray Diffraction (WAXD) of p-WCT, MCNTs, C-MCNT0, C-MCNT0.5, C-MCNT1.0, C-MCNT2.0, C-MCNT3.0, and C-MCNT4.0

X-ray diffraction (XRD) patterns of p-WCT, MCNTs and cellulose/MCNT composite films were recorded with an X-ray diffractometer (D8 ADVANCE, Bruker, Germany), where 40 kV, 40 mA, 20 °/min, and CuKa (λ = 1.5406 Å) radiation were applied for all samples. XRD patterns of 2θ span in the range of 5–60° were output.

#### 2.3.3. The Wettability of C-MCNT0, C-MCNT0.5, C-MCNT1.0, C-MCNT2.0, C-MCNT3.0, and C-MCNT4.0

The wettability of the cellulose/MCNT composite films was evaluated via OCA 50 machine (Dataphysics, Germany). Seven positions were measured for each film, and the averaged value of contact angle was output.

#### 2.3.4. Fourier-Transform Infrared (FTIR) Spectra of p-WCT, MCNTs, C-MCNT0, C-MCNT0.5, C-MCNT1.0, C-MCNT2.0, C-MCNT3.0, and C-MCNT4.0

The difference in structure of the regenerated cellulose/MCNT films (C-MCNT0, C-MCNT0.5, C-MCNT1.0, C-MCNT2.0, C-MCNT3.0, and C-MCNT4.0) and their raw materials (p-WCT and MCNTs) was evaluated via Fourier-transform infrared spectrometer (Bruker, Germany) in attenuated-total-reflection (ATR) mode. A resolution of 4 cm^−1^ and 32 scans were used.

#### 2.3.5. Micro-Structures of the Cellulose/MCNT Films

Micro-structures of cellulose/MCNT composite films were evaluated using Korea COXEM EM-30 Plus scanning electron micrographs. Film samples were quenched in liquid nitrogen to see their cross-sectional micro-structures, and all samples were sprayed with a thin layer of gold before observation.

#### 2.3.6. Thermogravimetric Analysis (TGA) of p-WCT, MCNTs, C-MCNT0, C-MCNT0.5, C-MCNT1.0, C-MCNT2.0, C-MCNT3.0, and C-MCNT4.0

The thermal stability of p-WCT, MCNTs, and cellulose/MCNT composite films in nitrogen atmosphere was evaluated using a Q50 thermogravimetric analyzer (TA, United States). Heating speed of 15 °C/min and 5 mg film samples were used. The curves ranging from 50 °C to 800 °C were displayed.

#### 2.3.7. Mechanical Tests of C-MCNT0, C-MCNT0.5, C-MCNT1.0, C-MCNT2.0, C-MCNT3.0, and C-MCNT4.0

The mechanical performance of C-MCNT0, C-MCNT0.5, C-MCNT1.0, C-MCNT2.0, C-MCNT3.0, and C-MCNT4.0 films was characterized by TA.XT Plus C texture analyzer (Stable Micro System, UK). Each film was cut into rectangle shapes of 4.5 cm in length and 1.0 cm in width. The gauge length and drawing speed were set as 2.5 cm and 4.8 mm min^−1^.

## 3. Results and Discussion

### 3.1. Stability of Cellulose/MCNT Mixtures

As displayed in Figure 1a, homogeneous cellulose/MCNT mixtures were obtained via the in situ composition of MCNT dispersion and cellulose solution. It can be seen that MCNTs were dispersed evenly in AmimCl under mechanical stirring because of the strong interactions between AmimCl and MCNTs, which were similar to those in previous works [38,39]. Meanwhile, MCNTs were bounded tightly in the cellulose matrix by hydrogen bonding and physical entanglements (Figure 1b). Therefore, the cellulose/MCNT composite solutions could remain stable after preparation for more than 11 months and no sediment could be observed, as displayed in Figure 1c.

### 3.2. Transparency and Ultraviolet–Visible Spectra of Cellulose/MCNT Hydrogels

**Figure 2:**
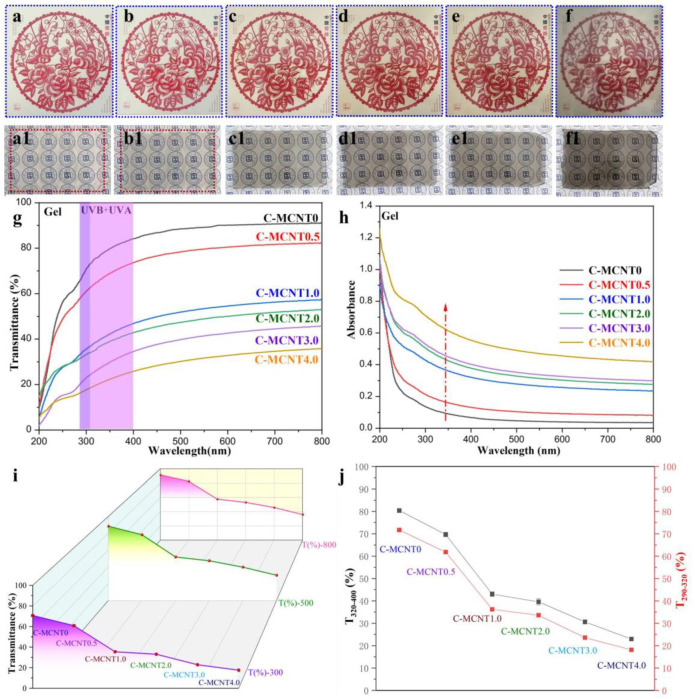
(**a**–**f**,**a1**–**f1**) Digital images of cellulose/MCNT hydrogels against different backgrounds ((**a**,**a1**) C-MCNT0, (**b**,**b1**) C-MCNT0.5, (**c**,**c1**) C-MCNT1.0, (**d**,**d1**) C-MCNT2.0, (**e**,**e1**) C-MCNT3.0, (**f**,**f1**) C-MCNT4.0); transmittance (**g**) and absorbance (**h**) of cellulose/MCNT composite hydrogels; (**i**) T(%)-800, T(%)-500, and T(%)-300 curves of cellulose/MCNT composite hydrogels; (**j**) T_320–400_ and T_290–320_ of cellulose/MCNT composite hydrogels.

Figure 2a–f,a1–f1 show the digital images of cellulose/MCNT hydrogels against Chinese paper cuttings and our university logo. It can be seen that C-MCNT0 hydrogels made from pretreated waste cotton textiles (p-WCTs) are colorless and transparent, which is common for native cellulose films without any additives, indicating the good quality of p-WCTs, which is consistent with our previous work [41]. Meanwhile, the cellulose/MCNT hydrogels display a dark color after the incorporation of MCNTs. Moreover, the dark color is strengthened by increasing the content of MCNTs in the composite hydrogels, and the C-MCNT4.0 gel is the darkest hydrogels. Figure 2f,f1 show that our university logo and the magpies covered by the C-MCNT4.0 hydrogel are still clear to see, although the content of MCNTs is as high as 4.0%. Moreover, MCNTs are dispersed evenly in the cellulose matrix. Additionally, the transmittance of cellulose/MCNT hydrogels is displayed in Figure 2g. It can be concluded that both the neat cellulose (C-MCNT0) hydrogel and C-MCNT0.5 hydrogel offer high transmittance, but the transmittance of cellulose/MCNT hydrogels is obviously decreased with the addition of more content of MCNTs. It is worth noticing that cellulose/MCNT composite hydrogels show improved ultraviolet-shielding properties, due to the unsaturated structure of carbon nanotubes, which is also demonstrated in Figure 2h. The T(%)-800, T(%)-500, and T(%)-300 curves of cellulose/MCNT composite hydrogels are also illustrated in Figure 2i to see the changing trends in the transparency and ultraviolet-shielding properties of cellulose/MCNT hydrogels. It can be noticed that the reduction in transmittance in the visible region (400–800 nm) is sharper than that in the ultraviolet region (200–400 nm), but the C-MCNT4.0 hydrogel still offers good transparency. Meanwhile, the average transmittance in the ranges of 320–400 nm (T_320–400_) and 290–320 nm (T_290–320_) are also calculated and adopted to judge the ultraviolet-shielding properties of the cellulose/MCNT hydrogels in Figure 2j. The obvious declines in T_320–400_ and T_290–320_ lead to the enhanced anti-ultraviolet capacity of the cellulose/MCNT composite hydrogels. To summarize, it has been proven that the ultraviolet-shielding capacity of the hybrid hydrogels is significantly improved by increasing the content of MCNTs.

### 3.3. Transparency and Ultraviolet–Visible Spectra of Cellulose/MCNT Films

**Figure 3:**
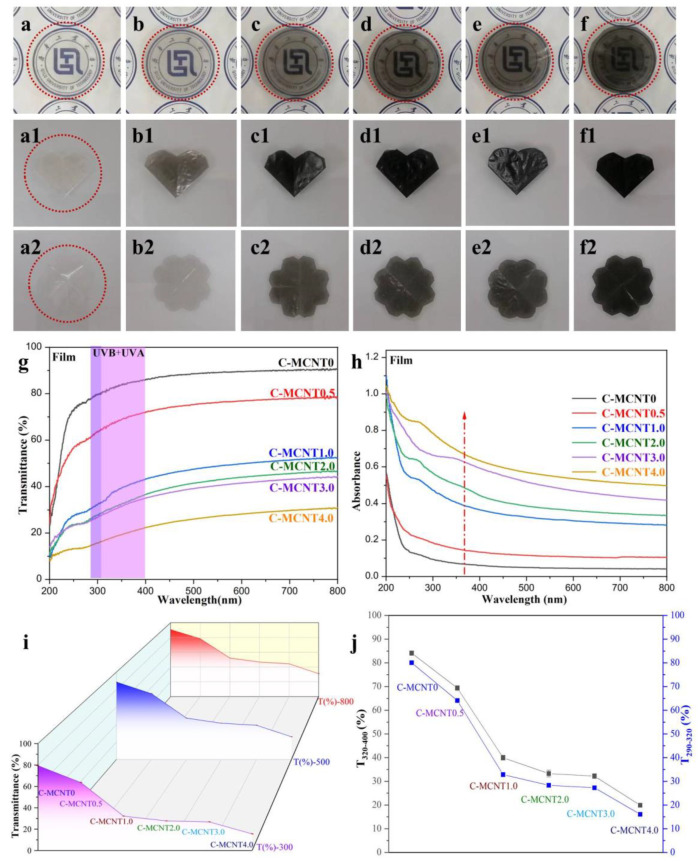
Digital images of cellulose/MCNT films against our university logos (**a**–**f**), folded into heart shapes (**a1**–**f1**) and unfolded (**a2**–**f2**). ((**a**,**a1**,**a2**) C-MCNT0; (**b**,**b1**,**b2**) C-MCNT0.5; (**c**,**c1**,**c2**) C-MCNT1.0; (**d**,**d1**,**d2**) C-MCNT2.0; (**e**,**e1**,**e2**) C-MCNT3.0; (**f**,**f1**,**f2**) C-MCNT4.0); Transmittance (**g**) and absorbance (**h**) of cellulose/MCNT composite films; (**i**) T(%)-800, T(%)-500, and T(%)-300 curves of cellulose/MCNT composite films; (**j**) T_320–400_ and T_290–320_ of cellulose/MCNT composite films.

Digital images of the cellulose/MCNT films against our university logos are shown in Figure 3a–f. Similarly to cellulose/MCNT hydrogels, the neat cellulose (C-MCNT0) film is colorless, and cellulose/MCNT composite films grow darker with an increase in the content of MCNTs. In contrast, the dark color of cellulose/MCNT films is strengthened when they are dried from their corresponding hydrogels. Herein, the background is difficult to see clearly when it is covered by the C-MCNT4.0 composite film. Nevertheless, the dispersion of MCNTs is still even, due to the stability of the cellulose/MCNT mixture and the homogeneity of cellulose/MCNT hydrogels. Additionally, the cellulose/MCNT films demonstrate good flexibility. All the films can be folded two times into heart shapes (Figure 3(a1–f1)) and unfolded again to their original flower shapes (Figure 3(a2–f2)), which is also one of the key indicators for their wrapping and packaging applications.

Additionally, Figure 3g demonstrates the transmittance of cellulose/MCNT composite films. It can be seen that the transmittance of neat cellulose films without any additives can reach 90.7% at a wavelength of 800 nm in the visible light region, indicating their good transparency. Meanwhile, the C-MCNT0.5 film still shows the relative high transmittance of 78.34%. Fortunately, the transmittance decreased sharply for the C-MCNT1.0 (52.23%), C-MCNT2.0 (46.79%), C-MCNT3.0 (44.41%), and C-MCNT4.0 (31.03%) films. Additionally, cellulose/MCNT composite films offer enhanced UV-shielding performance derived from their hydrogels, as displayed in Figure 3h. Nevertheless, the reduction in transmittance in the visible region (400–800 nm) is greater than that in the ultraviolet region (200–400 nm), which can be seen from the curves of T(%)-800, T(%)-500, and T(%)-300 displayed in Figure 3i. These results are comparable with our previous works [11,18]. Furthermore, the improved ultraviolet-shielding capacity is obviously exhibited in Figure 3j, where the averaged transmittance in the range of 320–400 nm (T_320–400_) and 290–320 nm (T_290–320_) of cellulose/MCNT composite films is calculated and output. It can be concluded that T_320–400_ and T_290–320_ of the regenerated films are also obviously reduced with increasing MCNT content. Shortly, the changing trends in the transparency and UV-Vis spectra of cellulose/MCNT films are similar to those of cellulose/MCNT composite hydrogels, indicating that the structures and properties of cellulose/MCNT films depend directly on their hydrogels. Therefore, both anti-ultraviolet cellulose-based gels and films could be obtained via the addition of anti-ultraviolet MCNT additives in this work.

### 3.4. FTIR, XRD, and TGA of Cellulose/MCNT Films

**Figure 4:**
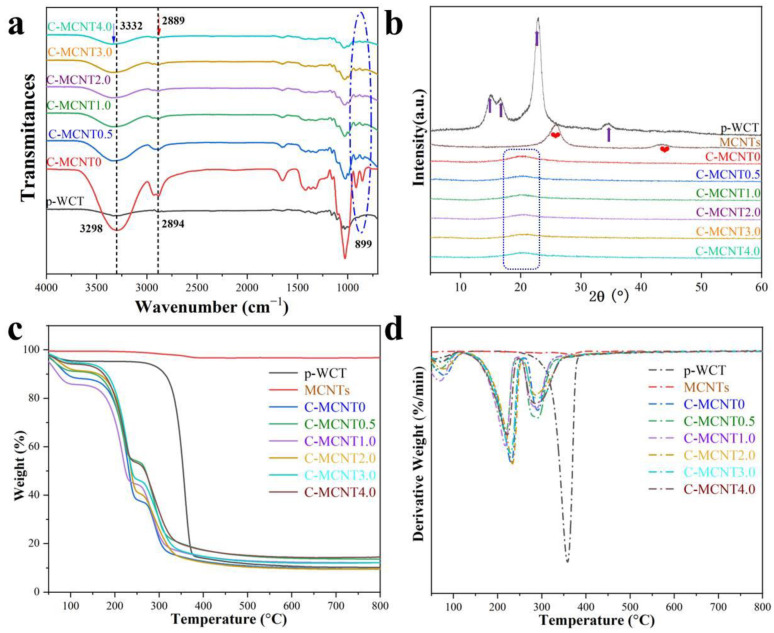
(**a**) Fourier transform infrared spectroscopy (FTIR), (**b**) X-ray diffractograms (XRDs), (**c**) thermogravimetric (TG) curves, and (**d**) derivative thermogravimetric (DTG) curves of the cellulose/MCNT films and their raw materials.

The FTIR curves of the regenerated cellulose/MCNT films (C-MCNT0, C-MCNT0.5, C-MCNT1.0, C-MCNT2.0, C-MCNT3.0, and C-MCNT4.0) and p-WCT are output to compare the difference in their structures, as displayed in Figure 4a. Similar with previous works, AmimCl is the non-derivative solvent for cellulose and, hence, no new functional groups appear in the FTIR spectra of the C-MCNT0 films [11,18,41,42]. Meanwhile, the regenerated cellulose/MCNT composite films show similar spectra to that of the C-MCNT0 film, indicating that cellulose and MCNTs are physically mixed and no chemical reactions occur between them during the preparation of the composite films. Generally, there is a crystal phase transformation after the cellulose dissolution and regeneration processes. Meanwhile, the C-H stretching band ranging from 2700 cm^−1^ to 3000 cm^−1^ and the O-H stretching band ranging from 3000 cm^−1^ to 3700 cm^−1^ are quite sensitive to this change [11]. Hence, the O-H stretching and C-H stretching bands for the p-WCT are at 3298 cm^−1^ and 2894 cm^−1^, respectively. For the regenerated films, the O-H stretching band moves to the higher wavelength and the C-H stretching band shifts to the lower wavelength. Particularly, the O-H stretching peak and C-H stretching peak for the C-MCNT4.0 composite film are at 3332 cm^−1^ and 2889 cm^−1^, respectively. Additionally, the absorption peak at 899 cm^−1^ is weak for p-WCT, but it enhanced intensively in spectra of C-MCNT0, C-MCNT0.5, C-MCNT1.0, C-MCNT2.0, C-MCNT3.0, and C-MCNT4.0 composite films, proving that the structure and hydrogen-bonding change significantly after the regeneration process, which is also in correspondence with previous works [18].

X-ray diffractograms of the C-MCNT0, C-MCNT0.5, C-MCNT1.0, C-MCNT2.0, C-MCNT3.0, and C-MCNT4.0 composite films and their raw materials (p-WCT and MCNTs) are shown in Figure 4b. Clearly, p-WCT shows obvious peaks at around 2θ = 16.5° (110), 22.5° (200), 34.5° (004), and 15.0° (1–10), which are characteristic peaks of I phase cellulose [18,41]. In contrast, these peaks disappear completely for the regenerated films. Moreover, C-MCNT0, C-MCNT0.5, C-MCNT1.0, C-MCNT2.0, C-MCNT3.0, and C-MCNT4.0 films only show a broad band at approximately 2θ = 20.0°, ascribed to the merged peaks of II phase and amorphous cellulose [11,18], indicating that the cellulose structure changes greatly, which is in correspondence with the previous FTIR results. Meanwhile, MCNTs display distinct peaks at around 26.0° and 43.0°, but these two peaks cannot be seen in the curves of cellulose/MCNT films, which may be attributed to the lower content of MCNTs. Additionally, the C-MCNT0, C-MCNT0.5, C-MCNT1.0, C-MCNT2.0, C-MCNT3.0, and C-MCNT4.0 composite films show similar curves, suggesting that the addition of MCNTs has no obvious effects on the crystallization of the cellulose in this work.

The thermal properties of the cellulose/MCNT (C-MCNT0, C-MCNT0.5, C-MCNT1.0, C-MCNT2.0, C-MCNT3.0, and C-MCNT4.0) films and their raw materials are compared by analyzing their thermogravimetric (TG) and derivative thermo-gravimetric data (DTG) curves (Figure 4c,d). Firstly, the mass loss below 120 ℃ of all samples is ascribed to the loss of moisture [11,18]. It can be seen that p-WCT contains a higher moisture content than MCNTs. Moreover, cellulose/MCNT films have a highest moisture content because glycerol was added in the last coagulation and more water is retained in the films, as displayed in Figure 4d. Hence, all cellulose/MCNT films display the second mass loss peak at around 225 °C in their DTG curves, which is attributed to the loss of glycerol, while the TG and DTG curves of p-WCT and MCNTs are smooth at this temperature. It is worth noticing that MCNTs are very stable during the heating process and they only show a small peak at around 370 °C. Additionally, the maximum weight loss rate (T_max_) of p-WCT is as high as about 360 °C, while the last peaks in the DTG curves of all cellulose/MCNT films range from 280 °C to 300 °C, suggesting a decrease in cellulose stability after the regeneration process. In short, the content of MCNTs shows some effects on the thermal stability of cellulose/MCNT films, but the changing trend in the last T_max_ of cellulose/MCNT films is not regular.

### 3.5. Mechanical Properties and Wettability of Cellulose/MCNT Hybrid Films

**Figure 5:**
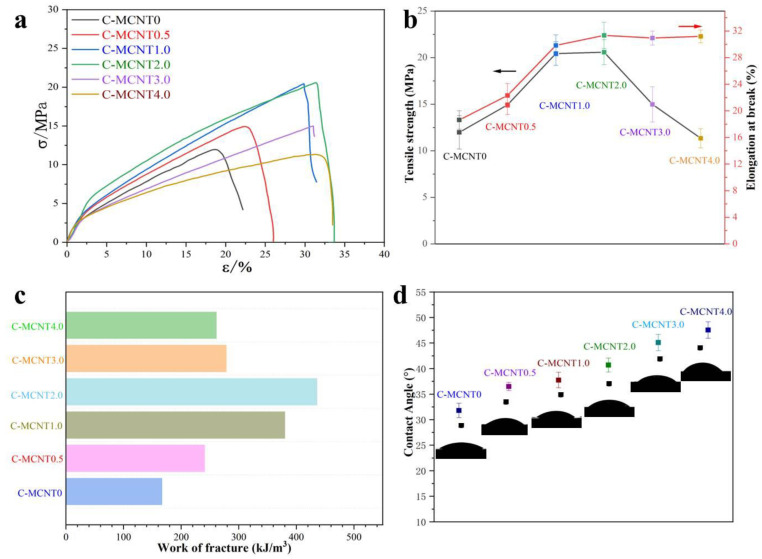
Stress–strain curves (**a**), tensile strength and elongation at break (**b**), work of fracture (**c**), and surface wettability (**d**) of the cellulose/MCNT composite films.

As we know, mechanical performance is the most important indicator for the utilization of composite films, and the mechanical performances of the C-MCNT0, C-MCNT0.5, C-MCNT1.0, C-MCNT2.0, C-MCNT3.0, and C-MCNT4.0 composite films are shown in Figure 5a–c. The tensile strength of C-MCNT0 prepared from the pretreated waste cotton textiles is about 11.97 MPa, indicating the high quality of p-WCTs. Meanwhile, the dissolution of cellulose in ionic liquids is gentle, which is also beneficial in protecting the tensile strength of the regenerated cellulose material. Moreover, the tensile strength of regenerated cellulose films can be enhanced via the incorporation of nano-fillers, such as aramid nanofibers (ANFs) [18]. Hence, the tensile strength of the C-MCNT0.5 film is increased to 14.93 MPa, 24.75% higher than that of neat cellulose films (C-MCNT0). Moreover, the tensile strengths of C-MCNT1.0 and C-MCNT2.0 can reach as high as 20.44 MPa and 20.58 MPa, respectively. Fortunately, the tensile strengths of cellulose/MCNT composite films are decreased with the increased addition of MCNTs, where the tensile strengths of C-MCNT3.0 and C-MCNT4.0 composite films are 14.99 MPa and 11.34 MPa, indicating that nano-fillers with high content trend to aggregate in the matrix. As we know, most cellulose-based films are brittle and the elongations at breaks are generally below 6% without any incorporation [41,42]. However, plasticizers like glycerol can solve this problem, and the elongations at breaks of C-MCNT0, C-MCNT0.5, C-MCNT1.0, C-MCNT2.0, C-MCNT3.0, and C-MCNT4.0 composite films are 18.63%, 22.32%, 29.85%, 31.35%, 30.95%, and 31.20%, respectively, which are higher than that of commercial cellophane. Additionally, the toughness of the cellulose/MCNT composite films can be evaluated via the work of fracture, the integral area of stress–strain curves [18]. The works of fracture of C-MCNT0, C-MCNT0.5, C-MCNT1.0, C-MCNT2.0, C-MCNT3.0, and C-MCNT4.0 composite films are 166.74 MJ/m^3^, 240.59 MJ/m^3^, 379.88 MJ/m^3^, 435.84 MJ/m^3^, 278.10 MJ/m^3^, and 261.12 MJ/m^3^, meaning that the composite film has good toughness. Herein, the mechanical properties of cellulose/MCNT composite films are good enough to be used as wrapping and packaging materials and the addition of MCNTs has an obvious effect on their mechanical properties.

The water contact angles of the C-MCNT0, C-MCNT0.5, C-MCNT1.0, C-MCNT2.0, C-MCNT3.0, and C-MCNT4.0 composite films are displayed in Figure 4d to evaluate their surface wettability. As we know, native cellulose film is hydrophilic. Herein, the water contact angle of C-MCNT0 made from raw p-WCTs is 31.80°. Generally, unmodified MCNTs are hydrophobic. Thus, the water contact angles of the cellulose/MCNT films are increased with the content of MCNTs rising from 0.5% to 4.0%. The water contact angles of C-MCNT0.5, C-MCNT1.0, C-MCNT2.0, C-MCNT3.0, and C-MCNT4.0 composite films are about 36.5°, 37.75°, 40.7°, 45.1°, and 47.55°, respectively. As a result, the content of MCNTs shows obvious effects on the wettability of cellulose/MCNT films.

### 3.6. Micro-Structures of the Cellulose/MCNT Films

**Figure 6:**
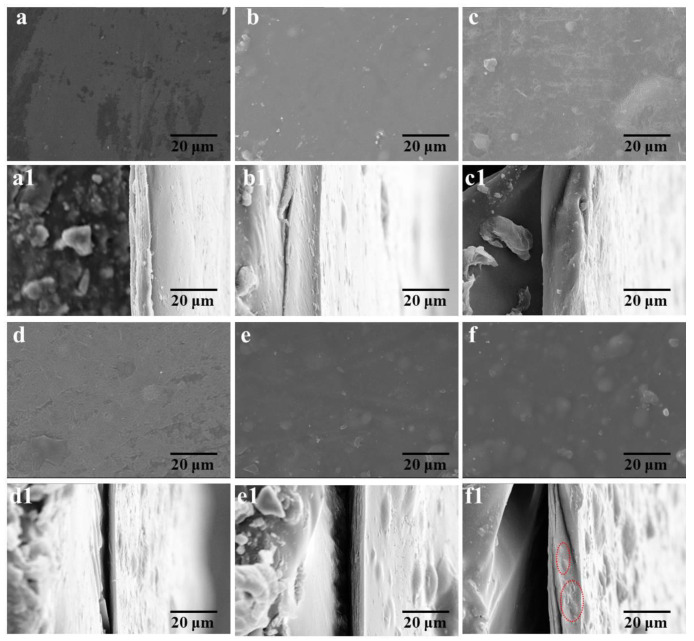
(**a**–**f**,**a1**–**f1**) SEM micro-images of the cellulose/MCNT films: (**a**–**f**) the surface photographs of the cellulose/MCNT films; (**a1**–**f1**) the cross-section photographs of the cellulose/MCNT films.

The cross-sectional and surface micro-structures of C-MCNT0, C-MCNT0.5, C-MCNT1.0, C-MCNT2.0, C-MCNT3.0, and C-MCNT4.0 composite films are recorded, as demonstrated in Figure 6. The surface micro-images of the cellulose/MCNT films are shown in Figure 6a–f, and all cellulose/MCNT films have relatively smooth surfaces, except that some minor impurities are stuck on the surfaces during the preparing process. It can be noticed that the surfaces of C-MCNT3.0 and C-MCNT4.0 films become rougher when the content of MCNTs is above 2.0%, meaning that MCNTs with high content may become drawbacks in composite films. Additionally, C-MCNT0, C-MCNT0.5, C-MCNT1.0, and C-MCNT2.0 composite films also display dense and homogeneous cross-sectional micro-structures, as shown in Figure 6a1–d1. Nevertheless, there are some tiny clusters in the C-MCNT3.0 and C-MCNT4.0 films (Figure 6e1–f1), indicating the aggregation of MCNTs, which were also mentioned in previous works [11,18]. Therefore, the micro-structures of cellulose/MCNT films are influenced significantly by the content of MCNTs.

## 4. Conclusions

By using cotton textile waste as a raw material, homogeneous and transparent cellulose/MCNT hybrid gels and films with improved ultraviolet-shielding properties were manufactured successfully via sol–gel technology, and MCNTs could be dispersed evenly in cellulose without any chemical and physical pretreatments. Cellulose/MCNT hybrids showed uniform structures due to the intensive interactions between cellulose and MCNTs. Nevertheless, no chemical reactions were found during the preparation of cellulose/MCNT gels and films. It was found that the structures and performance of composites were obviously influenced by the content of MCNTs. T_320–400_ and T_290–320_ of cellulose/MCNT composite gels and films decreased sharply with an increase in the addition of MCNTs, meaning that ultraviolet-shielding properties were given to cellulose/MCNT gels and films without significantly sacrificing their transparency. Specifically, the T_320–400_ of C-MCNT4.0 composite gel and film was 22.93% and 19.91%, and their T_290–320_ was 18.81% and 16.09%, much lower than those of the neat cellulose C-MCNT0 gel (T_320–400_: 80.41% and T_290–320_: 71.71%) and film (T_320–400_: 84.12% and T_290–320_: 80.03%). Meanwhile, the incorporation of MCNTs decreased the hydrophilicity of the cellulose film, and the WCA of the C-MCNT4.0 composite film was about 47.55°, 49.52% higher than that of the C-MCNT 0 film (31.80°). Hence, more content of MCNTs is required to obtain cellulose/MCNT gels and films with low hydrophilicity and high ultraviolet-shielding properties. Additionally, additives such as glycerol and MCNTs played an important role in the mechanical performance of cellulose/MCNT composite films. Glycerol acted as a plasticizer to improve the elongations at break of the cellulose/MCNT films, while the MCNTs elevated their tensile strength when the content of MCNTs was below 2%. It is worth noticing that the maximum tensile strength of cellulose/MCNT (C-MCNT2.0) film was around 20.58 MPa and the elongation at break was approximately 31.35%, meaning that it showed great potential for use in packaging materials. Therefore, 2% of MCNTs was recommended to obtain cellulose/MCNT films with good mechanical properties. In short, in this work, we have reported a simple and effective approach to dispersing MCNTs evenly in a cellulose matrix, and cotton textile waste was employed to prepare transparent and anti-ultraviolet cellulose/MCNT hybrid gels and films simultaneously, which is beneficial to our ecological environment and allows the utilization of natural resources.

## Figures and Tables

**Table 1:** The chemical content and proportion of cellulose/MWCT films.

Sample	Cellulose (%)	MCNTs (%)
C-MCNT0	100	0
C-MCNT0.5	99.5	0.5
C-MCNT1.0	99.0	1.0
C-MCNT2.0	98.0	2.0
C-MCNT3.0	97.0	3.0
C-MCNT4.0	96.0	4.0

## Data Availability

Data are contained within the article.
